# Axin downregulates TCF-4 transcription via β-catenin, but not p53, and inhibits the proliferation and invasion of lung cancer cells

**DOI:** 10.1186/1476-4598-9-25

**Published:** 2010-02-02

**Authors:** Lian-He Yang, Hong-Tao Xu, Yang Han, Qing-Chang Li, Yang Liu, Yue Zhao, Zhi-Qiang Yang, Qian-Ze Dong, Yuan Miao, Shun-Dong Dai, En-Hua Wang

**Affiliations:** 1 Department of Pathology, The First Affiliated Hospital and College of Basic Medical Sciences of China Medical University, Shenyang 110001, China

## Abstract

**Background:**

We previously reported that overexpression of Axin downregulates T cell factor-4 (TCF-4) transcription. However, the mechanism(s) by which Axin downregulates the transcription and expression of TCF-4 is not clear. It has been reported that β-catenin promotes and p53 inhibits TCF-4 transcription, respectively. The aim of this study was to investigate whether β-catenin and/or p53 is required for Axin-mediated downregulation of TCF-4.

**Results:**

Axin mutants that lack p53/HIPK2 and/or β-catenin binding domains were expressed in lung cancer cells, BE1 (mutant p53) and A549 (wild type p53). Expression of Axin or AxinΔp53 downregulates β-catenin and TCF-4, and knock-down of β-catenin upregulates TCF-4 in BE1 cells. However, expression of AxinΔβ-ca into BE1 cells did not downregulate TCF-4 expression. These results indicate that Axin downregulates TCF-4 transcription via β-catenin. Although overexpression of wild-type p53 also downregulates TCF-4 in BE1 cells, cotransfection of p53 and AxinΔβ-ca did not downregulate TCF-4 further. These results suggest that Axin does not promote p53-mediated downregulation of TCF-4. Axin, AxinΔp53, and AxinΔβ-ca all downregulated β-catenin and TCF-4 in A549 cells. Knock-down of p53 upregulated β-catenin and TCF-4, but cotransfection of AxinΔβ-ca and p53 siRNA resulted in downregulation of β-catenin and TCF-4. These results indicate that p53 is not required for Axin-mediated downregulation of TCF-4. Knock-down or inhibition of GSK-3β prevented Axin-mediated downregulation of TCF-4. Furthermore, expression of Axin and AxinΔp53, prevented the proliferative and invasive ability of BE1 and A549, expression of AxinΔβ-ca could only prevented the proliferative and invasive ability effectively.

**Conclusions:**

Axin downregulates TCF-4 transcription via β-catenin and independently of p53. Axin may also inhibits the proliferation and invasion of lung cancer cells via β-catenin and p53.

## Background

The wingless/int (Wnt) signaling pathway plays an important role in tumor cell de-differentiation and proliferation [1]. Evidence indicates that abnormal activation of the Wnt pathway plays an important role in tumor progression [2-5]. Activation of the Wnt pathway requires nuclear accumulation of β-catenin, as well as association of β-catenin and T cell factor-4 (TCF-4). TCF-4 induces transcription of target genes, such as *c-myc*, *cyclin D1*, vascular endothelial growth factor (*vegf*) and matrix metalloproteinase-7 (*mmp-7*) [1,6]. Under normal circumstances, GSK-3β phosphorylates excess β-catenin facilitating its degradation, resulting in the dynamic balance between β-catenin generation and degradation. Mutations resulting in constitutive nuclear accumulation of β-catenin leads to abnormal Wnt pathway signaling causing tumor formation [4].

Axin, the product of the mouse *Fused (Fu) *gene, was originally identified as an inhibitor of the Wnt-signaling pathway by virtue of its ability to regulate embryonic axis formation [7,8]. Axin inhibits the Wnt pathway by facilitating degradation of β-catenin by serving as a scaffolding protein enabling assembly of the APC/Axin/GSK-3β/β-catenin complex [9]. The Axin antagonist, Dishevelled, positively regulates the Wnt signaling pathway by binding to Axin directly [10,11], and prevents inhibition of GSK-3β-dependent phosphorylation of β-catenin. Most likely this occurs through dissociation of the APC/Axin/GSK-3β complex [11,12].

Since the Wnt pathway plays an important role in tumor cell de-differentiation, proliferation and tumor progression, its primary negative regulator, Axin, is recognized as an important tumor suppressor [13]. Our previous study demonstrated that decreased Axin expression correlates with nuclear localization of β-catenin and poor differentiation of lung cancer cells. Expression of Axin not only induced β-catenin degradation, but also significant downregulation of TCF-4 mRNA and protein expression. Expression of Axin resulted in decreased proliferation and invasion of lung cancer cells [5]. A mechanism by which Axin downregulates the transcription of TCF-4 has not been described to date. It has been reported that overexpression of p53 can downregulate TCF-4 transcription, but p53 do not interact with TCF-4 promoter directly which indicated that some p53 downstream genes downregulate TCF-4 transcription [14]. Futhermore, Axin could induce p53-dependent transcriptional activity [15], so it seems likely that Axin might be able to downregulate TCF-4 mRNA via p53 activation. In other words, Axin-p53-TCF-4 may act as an alternative for Axin-β-catenin-TCF-4 when wild type p53 is present. β-catenin upregulates TCF-4 transcript and protein expression [16]. Therefore, we propose that Axin may downregulate TCF-4 expression and TCF-mediated gene transcription via activation of the p53 pathway and/or facilitation of β-catenin degradation.

In order to test this hypothesis, we expressed three mutant Axin plasmids, which inhibit the interaction of Axin with β-catenin and/or p53 in lung carcinoma cell lines, BE1 (mutant p53) and A549 (wild type p53) [17]. We hypothesize that Axin expression downregulates TCF-4 transcription and TCF-mediated gene transcription activity in a β-catenin- and/or p53-dependent manner in lung cancer cells.

## Results

### Axin and AxinΔp53 downregulate TCF-4 expression and TCF-mediated gene transcription activity in BE1 cells

**Figure 1:**
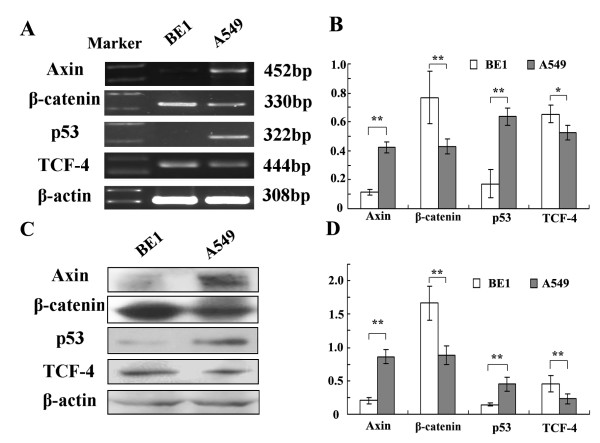
**The mRNA and protein expression levels of Axin and other factors in BE1 and A549 cell line**. (A) RT-PCR analysis of BE1 and A549 cells using the indicated specific primers. (B) Quantitative representation of the RT-PCR analysis. (C) Western blot analyses of BE1 and A549 cells using specific antibodies to the indicated proteins. (D) Quantitative representation of the Western blot anlaysis. * *P *< 0.05, ** *P *< 0.01.

We first detected the basal expression levels of Axin, β-catenin, p53, and TCF-4 in BE1 and A549 cells. The Axin and p53 proteins are barely detectable in BE1 cells. In addition, *p53 *is mutant and mRNA levels are low in BE1 cells [17]. However, Axin and p53 were moderately expressed in A549 cells. BE1 cells expressed more β-catenin and TCF-4 than A549 cells, both at the mRNA and protein levels (*P *< 0.01, Figure 1).

**Figure 2:**
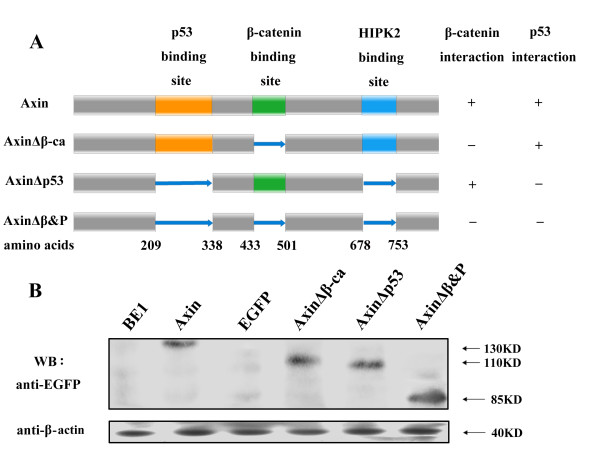
**The composition and expression of Axin mutants**. (A) Schematic representation of the mutants of the Axin mutants used in this study. The binding sites for HIPK2, β-catenin and p53 are indicated. (B) BE1 cells were transfected with the indicated EGFP-tagged Axin construct. Total cell lysates were resolved by SDS-PAGE and Western blot analysis was performed using anti-EGFP antibody to detect the expression of the different Axin mutants.

**Figure 3:**
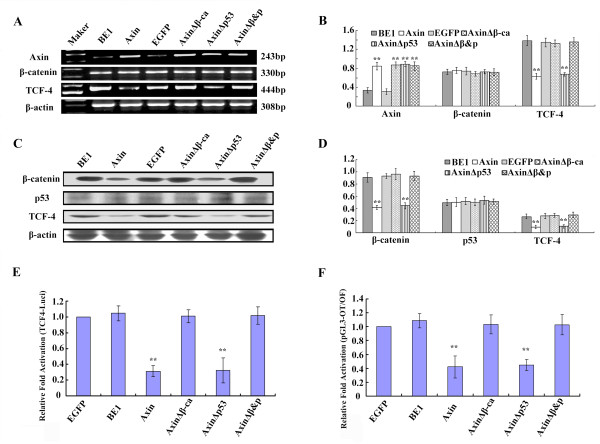
**The effect of transfecting various Axin plasmids into BE1 cells**. Axin, AxinΔβ-ca, AxinΔp53 and AxinΔβ&P were transfected into BE1 cells. (A) Forty-eight hours after transfection total RNA was isolated and RT-PCR analysis was performed using the indicated primers. (B) Quantification of mRNA expression after transfection. (C) Proteins expression of BE1 cells 48 h after transfection using the indicated antibodies. (D) Quantification of protein expression 48 h after transfection of BE1 cells with (E) the TCF-4 promoter construct and (F) TCF reporter construct. * *P *< 0.05, ** *P *< 0.01.

Axin, AxinΔβ-ca (cannot bind β-catenin), AxinΔp53 (cannot bind p53), AxinΔβ&P (cannot bind either β-catenin or p53) or control vector were transfected into BE1 cells (Figure 2A). Axin protein and mRNA expression levels were determined to confirm expression (Figure 2B, Figure 3A and 3B). β-catenin mRNA expression levels were similar in all groups (*P *> 0.05, Figure 3A and 3B). However, transfection of Axin and AxinΔp53 results in significant reduction of TCF-4 mRNA levels (*P *< 0.01, Figure 3A and 3B). These data demonstrate that Axin downregulates TCF-4 transcription levels independently of p53. Levels of TCF-4 mRNA and protein in AxinΔβ-ca or AxinΔβ&P expressing cells was similar to the control cells (*P *> 0.05, Figure 3A and 3B), suggesting that β-catenin is required for Axin-mediated downregulation of TCF-4 transcription.

Western blot analysis confirmed that expression of Axin and AxinΔp53 resulted in a significant reduction in TCF-4 and β-catenin protein levels (*P *< 0.01, Figure 3C and 3D). These results suggest that Axin downregulates the expression of TCF-4 at the mRNA level and β-catenin at the protein level in a p53-independent, β-catenin-dependent manner in BE1 cells.

Dual-luciferase assay results show that expression of Axin and AxinΔp53 significantly downregulate TCF-4 promoter activity and TCF-mediated gene transcription activity (*P *< 0.01, Figure 3E and 3F). In contrast, no significant difference was detected in AxinΔβ-ca and AxinΔβ&P expressing cells when compared with controls (*P *> 0.05). These results show that Axin inhibits the Wnt signaling pathway by downregulating β-catenin and TCF-4 expression levels in a p53-independent, β-catenin-dependent manner.

### Axin, AxinΔp53, and AxinΔβ-ca downregulate TCF-4 expression and TCF-mediated gene transcription activity in A549 cells

**Figure 4:**
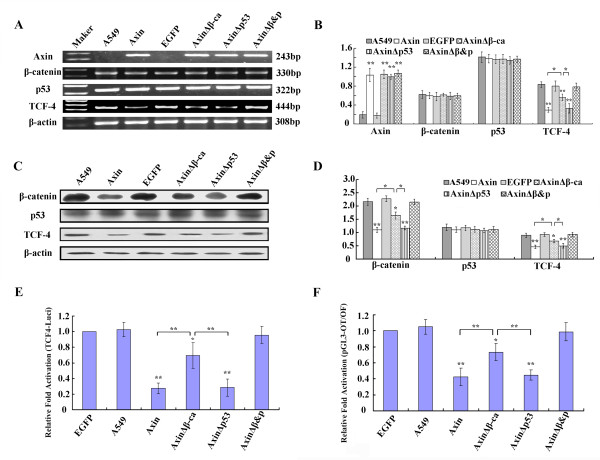
**The effect of transfecting various Axin plasmids into A549 cells**. Axin, AxinΔβ-ca, AxinΔp53 and AxinΔβ&P were transfected into A549 cells. (A) Forty-eight hours after transfection total RNA was isolated and RT-PCR analysis was performed using the indicated primers. (B) Quantification of mRNA expression after transfection. (C) Protein expression of A549 cells 48 h after transfection using the indicated antibodies. (D) Quantification of protein expression 48 h after transfection of A549 cells with (E) the TCF-4 promoter construct and (F) TCF reporter construct. * *P *< 0.05, ** *P *< 0.01.

To further delineate the mechanism by which Axin-mediates downregulation of TCF-4 transcription, we determined whether expression of wild type and/or mutant forms of Axin downregulate TCF-4 in cells with wild-type p53. A549 cells were transfected with Axin, AxinΔβ-ca, AxinΔp53 and AxinΔβ&P. Axin and Axin mutant mRNA expression levels were determined 48 h post-transfection to confirm expression (*P *< 0.01, Figure 4A and 4B). Protein expression was also confirmed (data not shown). There was no significant change in β-catenin and p53 mRNA levels in any group (*P *> 0.05). However, TCF-4 mRNA levels were significantly lower in Axin, AxinΔβ-ca and AxinΔp53 expressing cells compared to controls (*P *< 0.01, Figure 4A and 4B). It appears that Axin downregulates TCF-4 transcription via either β-catenin and p53 in A549 cells, as AxinΔβ&P expression does not result in downregulation of TCF-4 mRNA expression. In addition, we found that downregulation of TCF-4 mRNA in Axin and AxinΔp53 expressing cells was more significant than in AxinΔβ-ca expressing cells (*P *< 0.05). These data demonstrate that the β-catenin pathway is the primary mechanism by which Axin downregulates TCF-4 transcription.

Expression of Axin and AxinΔp53 in A549 cells results in significant downregulation of β-catenin and TCF-4 protein levels (*P *< 0.01, Figure 4C and 4D), similar to BE1 cells. In contrast to the results with BE1 cells, expression of AxinΔβ-ca also downregulated β-catenin and TCF-4 protein (*P *< 0.05), but the effect was significantly weaker than with expression of Axin and AxinΔp53 (*P *< 0.05). Expression of a mutant that cannot bind β-catenin or p53, AxinΔβ&P, resulted in no downregulation of TCF-4 mRNA or protein expression.

Dual-luciferase assay showed that expression of Axin, AxinΔp53 and AxinΔβ-ca in A549 cells leads to significant downregulation of TCF-4 promoter activity and TCF-mediated gene transcription (*P *< 0.01, Figure 4E and 4F). In contrast to BE1 cells, transfection of AxinΔβ-ca also downregulates TCF-4 promoter activity and TCF-mediated transcription (*P *< 0.05), but the effect was significantly weaker than with transfection of Axin or AxinΔp53 (*P *< 0.01).

### LiCl treatment and GSK-3β knock-down prevents Axin-mediated downregulation of TCF-4 in BE1 and A549 cells

**Figure 5:**
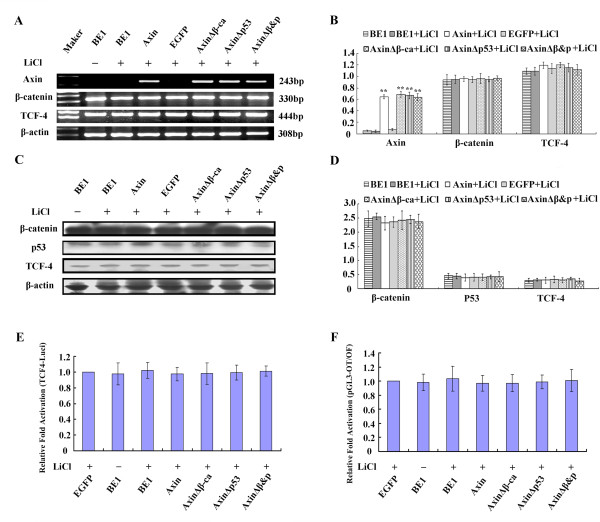
**The effect of LiCl treatment on BE1 cells transfected with different Axin plasmids**. Axin, AxinΔβ-ca, AxinΔp53 and AxinΔβ&P were transfected into BE1 cells. Twenty-four hours post-transfection, cells were treated with LiCl (20 mM). (A) Forty-eight hours after transfection total RNA and RT-PCR analysis was performed using the indicated primers. (B) Quantification of mRNA expression after transfection. (C) Protein expression in BE1 cells 48 h after transfection using the indicated antibodies. (D) Quantification of protein expression 48 h after transfection with (E) the TCF-4 promoter construct and (F) TCF reporter construct. * *P *< 0.05, ** *P *< 0.01.

To further investigate whether Axin downregulates TCF-4 via β-catenin, BE1 cells were transfected with wild-type and mutant Axin constructs, and, subsequently, treated with the GSK-3β inhibitor, LiCl. Expression levels of β-catenin and p53 mRNA did not change after treatment with LiCl for any transfection group (*P *> 0.05, Figure 5A and 5B). However, TCF-4 mRNA levels were not reduced in Axin and AxinΔp53 expressing cells after LiCl treatment compared to controls (*P *> 0.05).

**Figure 6:**
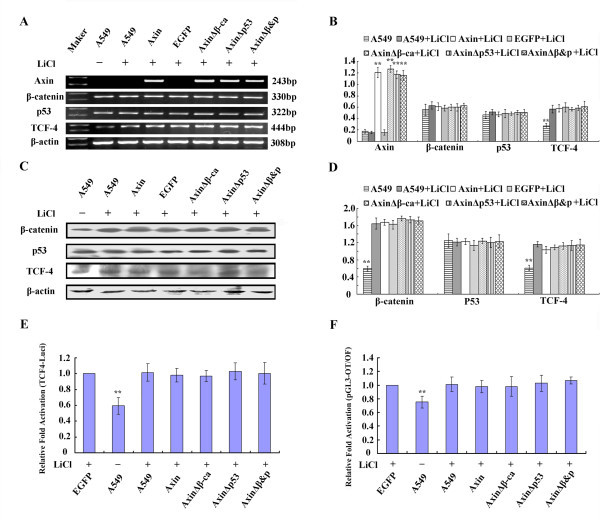
**The effect of LiCl treatment on A549 cells transfected with different Axin plasmids**. Axin, AxinΔβ-ca, AxinΔp53 and AxinΔβ&P were transfected into A549 cells. Twenty-four hours post-transfection, cells were treated with LiCl (20 mM). (A) Forty-eight hours after transfection total RNA and RT-PCR analysis was performed using the indicated primers. (B) Quantification of mRNA expression after transfection. (C) Protein expression in A549 cells 48 h after transfection using the indicated antibodies. (D) Quantification of protein expression 48 h after transfection with (E) the TCF-4 promoter construct and (F) TCF reporter construct. * *P *< 0.05, ** *P *< 0.01.

Western blot analysis shows that LiCl treatment of Axin and AxinΔp53 expressing BE1 cells prevents reduction of β-catenin and TCF-4 protein levels (*P *> 0.05, Figure 5C and 5D). These data suggest that TCF-4 mRNA expression levels are correlated with β-catenin protein expression levels in BE1 cells. Dual-luciferase assay demonstrates that LiCl treatment of Axin and AxinΔp53 expressing cells also inhibits downregulation TCF-4 promoter activity and TCF-mediated gene transcription activity (*P *> 0.05, Figure 5E and 5F). Collectively, these results suggest that upregulation of β-catenin and TCF-4 is required for increased TCF-4 promoter activity and TCF-mediated gene transcription activity. Similar results were obtained in Axin, AxinΔβ-ca or AxinΔp53 expressing A549 cells with LiCl treatment. Axin, AxinΔβ-ca or AxinΔp53-mediated reduction of TCF-4 mRNA and protein expression, and β-catenin protein, was inhibited with LiCl treatment. LiCl treatment did not affect β-catenin and p53 mRNA expresssion levels (*P *> 0.05, Figure 6). It was noted that the β-catenin and TCF-4 protein levels were inversely regulated in AxinΔβ-ca expressing A549 cells. Therefore, it seems that regardless of p53 status, TCF-4 mRNA levels were correlated with β-catenin protein levels.

We also found that LiCl treatment inhibited the downregulation of TCF-4 promoter activity and TCF-mediated gene transcription in Axin, AxinΔβ-ca and AxinΔp53 expressing A549 cells (*P *> 0.05, Figure 6E and 6F). Therefore, these data demonstrate that Axin requires GSK-3β to inihibit TCF-4 and suggests that this mechanism is likely β-catenin-dependent.

**Figure 7:**
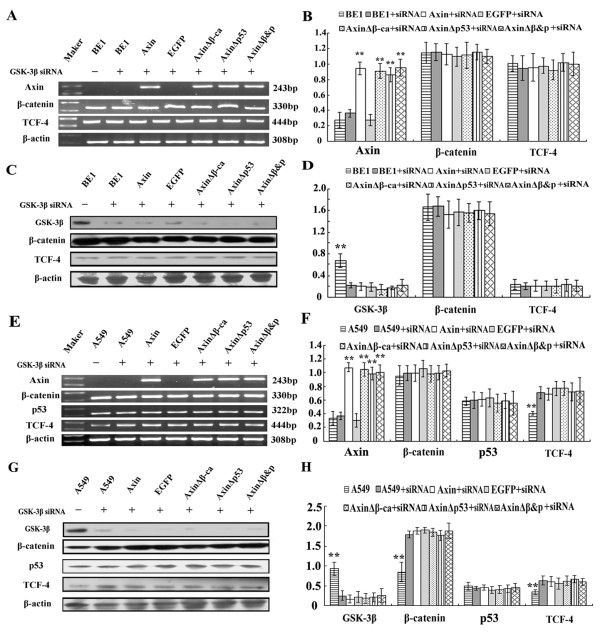
**The effect of GSK-3β siRNA treatment on BE1 cells and A549 cells transfected with different Axin plasmids**. Axin, AxinΔβ-ca, AxinΔP53 and AxinΔβ&P were cotransfected with GSK-3? siRNA into BE1 (A-D) and A549 (E-H) cells. (A&E) Forty-eight hours after transfection total RNA and RT-PCR analysis was performed using the indicated primers. (B&F) Quantification of mRNA expression after cotransfection. (C&G) Western blot analysis of BE1 and A549 cells 48 h after transfection using the indicated antibodies. (D&H) Quantification of protein expression after transfection. * *P *< 0.05, ** *P *< 0.01.

To confirm the LiCl treatment results, siRNA was used to knock-down GSK-3β to prevent β-catenin downregulation. Results were similar with LiCl treatment. Knock-down of GSK-3β inhibited Axin-mediated degradation of β-catenin and prevented downregulation of TCF-4 in both BE1 and A549 cells (*P *> 0.05, Figure 7).

### Wild-type p53 downregulates TCF-4 mRNA and protein, and is not enhanced by Axin

**Figure 8:**
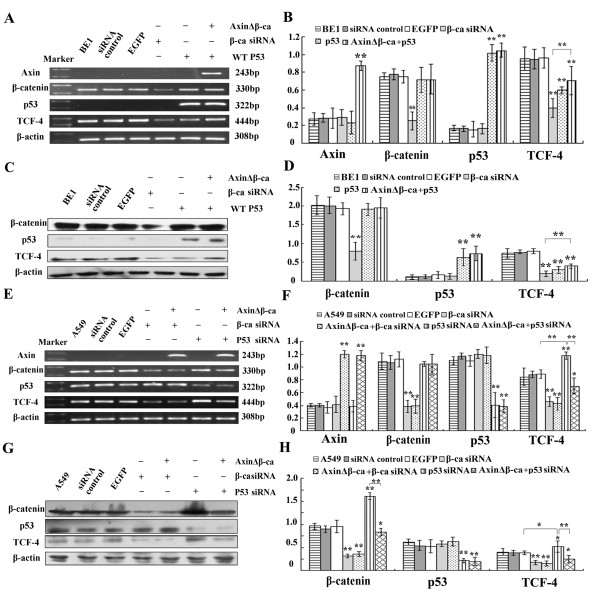
**Axin-mediated downregulation of TCF-4 is p53-independent**. BE1 cells were transfected with wild-type p53 or β-catenin siRNA, respectively, cotransfection of wild-type p53 and AxinΔβ-ca was also performed in BE1 cells. A549 cells were transfected with p53 siRNA or β-catenin siRNA, respectively, and cotransfection of β-catenin siRNA and AxinΔβ-ca, p53 siRNA and AxinΔβ-ca were also performed in A549 cells. (A&E) Forty-eight hours after transfection total RNA and RT-PCR analysis was performed using the indicated primers. EGFP and control siRNA served as control groups. (B&F) Quantification of mRNA expression after transfection. (C&G) Western blot analysis was performed 48 h after transfection using the indicated antibodies. (D&H) Quantification of protein expression after transfection * *P *< 0.05, ** *P *< 0.01.

To investigate whether AxinΔβ-ca downregulates TCF-4 transcription via p53, we transfected wild-type p53 into BE1 cells. Overexpression of p53 downregulates TCF-4 mRNA and protein, but cotransfection of AxinΔβ-ca and p53 did not downregulate TCF-4 further (Figure 8A-D). These results suggest that Axin cannot enhance p53-mediated downregulation of TCF-4, that Axin does not downregulate TCF-4 via p53, and that the Axin-p53-TCF-4 pathway is not functional in BE1 cells.

Furthermore, we downregulated p53 expression in A549 cells by siRNA. Results show that TCF-4 mRNA and protein were significantly upregulated after siRNA treatment, proving that wild-type p53 is able to downregulate TCF-4 (*P *< 0.01, Figure 8E-H). We next transfected AxinΔβ-ca into A549 cells in which p53 had been knocked-down, and found that AxinΔβ-ca further downregulate TCF-4 transcription, suggesting that Axin downregulates TCF-4 in a p53-independent manner.

To further investigate whether β-catenin expression positively correlates with TCF-4 expression, we knocked-down β-catenin in BE1 and A549 cells by siRNA. Loss of β-catenin resulted in downregulation of TCF-4 in both cell lines (*P *< 0.01, Figure 8). As we have already shown that Axin likely downregulates TCF-4 in a p53-independent manner. Transfection of AxinΔβ-ca into A549 cells did not result in further downregulation of TCF-4, suggesting that Axin downregulates TCF-4 via β-catenin, not p53.

### Axin reduces proliferation and invasion of lung cancer cells via β-catenin and p53

**Figure 9:**
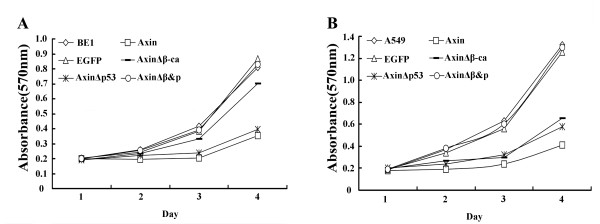
**Transfecting various Axin plasmids influences the proliferative ability of BE1 and A549 cells**. Cells were transfected with Axin, AxinΔβ-ca, AxinΔP53 and AxinΔβ&P and plated into wells 12 h post-transfection. MTT assay was performed every day for four days. Growth curves were generated for BE1 cells (A) and A549 cells (B).

To determine if Axin affects proliferation, cell growth assays were performed using Axin, AxinΔβ-ca, AxinΔp53 or AxinΔβ&P expressing BE1 and A549 cells. No marked difference was observed in BE1 cells expressing Axin and Axin mutant 24 h after transfection. However, 48 h post-transfection we found that expression of Axin and AxinΔp53 significantly decreased cellular proliferation of BE1 cells (*P *< 0.01, Figure 9A). Expression of AxinΔβ-ca only slightly decreased the growth of BE1 cells (*P *< 0.05). In A549 cells, expression of Axin, AxinΔp53 and AxinΔβ-ca significantly decreased proliferation at 48 h post-transfection (*P *< 0.01, Figure 9B). However, expression of Axin inhibited A549 cell proliferation more strongly than expression of AxinΔp53 or AxinΔβ-ca (*P *< 0.01). These results suggest that Axin inhibits proliferation of lung cancer cells via both the β-catenin and p53 pathways.

**Figure 10:**
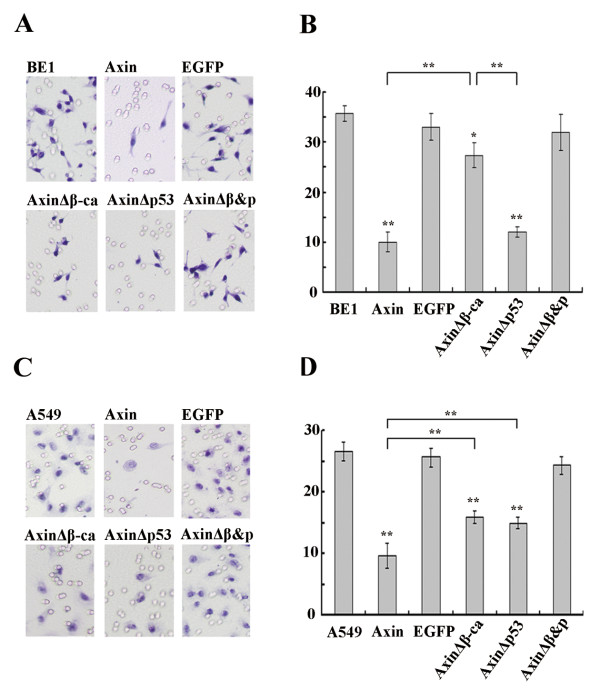
**Expression of various Axin constructs decreases the invasive ability of BE1 and A549 cells**. Cells were seeded on the upper chamber (5 × 10^4 ^cells/well) 24 h after transfection and incubated for 48 h. (A&C) A representative microscope field of filters under the Matrigel from BE1 and A549, respectively. (B&D) Quantification of the number of migrated BE1 and A549 cells, respectively. * *P *< 0.05, ** *P *< 0.01.

In order to determine if Axin plays a role in invasion, transwell assays were performed using Axin, AxinΔβ-ca, AxinΔp53 or AxinΔβ&P expressing BE1 and A549 cells. In transwell assays, invasion potential is measured by the number of cells that penetrate through the Matrigel layer and adhere to the transwell filter. Expression of Axin and AxinΔp53 in BE1 cells significantly decreased invasion compared with control groups by 48 h after transfection (*P *< 0.01, Figure 10A and 10B). Surprisingly, expression of AxinΔβ-ca also reduced the number of invasive cells (*P *< 0.05), but the effect was much weaker than with expression of Axin or AxinΔp53 (*P *< 0.01). These results suggest that the primary mechanism by which Axin reduces the invasion ability of lung cancer cells is through inhibition of β-catenin. Interestingly, in A549 cells, expression of AxinΔβ-ca decreased invasion as effectively as expression of AxinΔp53 (*P *< 0.01, Figure 10C and 10D), yet expression of Axin is the most potent inhibitor of invasion ability (*P *< 0.01). These results suggest that Axin decreases lung cancer cell invasion ability via the β-catenin and p53 pathways.

## Discussion

We have previously reported that there is a significant reduction in Axin expression and increased nuclear accumulation of β-catenin in human lung cancer specimens [18]. Axin decreased expression of TCF-4, but whether β-catenin is plats a role in this process has not been determined. Lin, *et al*. reported that Axin interacts with homeodomain-interacting protein kinase-2 (HIPK2) and p53, inducing p53 activity and facilitating p53-mediated transcription activity [15]. Rother, *et al*. found that some p53 target genes might downregulate TCF-4 transcription [14], so it seems likely Axin can downregulate TCF-4 transcription via p53. Others have reported that expression of β-catenin in endometrial cancer cells upregulates the expression of TCF-4 [16] and that Axin faciltates the degradation of β-catenin [9]. Therefore, it is also possible that Axin may downregulate TCF-4 transcription via β-catenin. But whether Axin downregulates TCF-4 via the p53 and/or β-catenin pathway and whether one pathway is preferred has not been determined. It has been shown by many *in vitro *studies that Axin interacts with GSK-3β, APC, and β-catenin to form a degradation complex. Furthermore, mutant Axin which cannot interact with GSK-3β, APC, or β-catenin cannot induce β-catenin degradation [19-25]. Therefore, we expressed wild type Axin and mutants lacking p53 and/or β-catenin binding domains in lung cancer cell lines expressing wild type (A549) or mutant (BE1) p53 to delineate the mechanism by which Axin downregulates TCF-4 expression.

Our results suggest that regardless of the status of the p53 pathway Axin is able to downregulate β-catenin protein, but not mRNA expression levels. In addition, Axin also downregulates TCF-4 expression levels and TCF-mediated gene transcription in a p53-independent manner. Furthermore, our results demonstrate that Axin-mediated downregulation of TCF-4 and TCF-mediated gene transcription is β-catenin dependent.

We have shown that an Axin construct that cannot bind β-catenin, AxinΔβ-ca, downregulates β-catenin and TCF-4. It has been previously reported that AxinΔβ-ca may still be effective in facilitating β-catenin destruction due to complementing interactions with other members of this complex [26]. However, we feel that this is not likely occurring in our experiments, because transfection of AxinΔβ-ca did not result in downregulation of TCF-4. Therefore, differences between our and previously published results might be caused by cell-type and/or species specific differences. The different results obtained from expression of AxinΔβ-ca in BE1 and A549 cells is likely caused by different endogenous Axin expression levels. Axin expression was barely detectable in BE1 cells, but moderate in A549 cells. It has been reported that dimerization of Axin is very important for its function [27]. So, it is possible that AxinΔβ-ca could form dimers with abundant wild-type Axin in A549 cells, making wild-type Axin more stable and effective, resulting in β-catenin degradation and TCF-4 downregulation in an Axin-β-catenin-TCF-4 manner. BE1 cells lack wild-type Axin, so AxinΔβ-ca could not function in the same manner. It has also been reported that Axin forms dimers by the DIX domain [28], and AxinΔβ-ca contains this domain. However, AxinΔβ&P also contains the DIX domain, but transfection of AxinΔβ&P did not result in downregulation of β-catenin and TCF-4. Furthermore, Lin, *et al. *found that Axin dimerization through the DIX domain is weak, and AxinΔβ&P Shearing three long parts that might influence the ability of AxinΔβ&P forming dimmers with wild type Axin, so transfection of AxinΔβ&P did not downregulate β-catenin and TCF-4 in A549.

We treated A549 by LiCl to block the degradation of β-catenin, we found that in A549 cells after LiCl treatment, the expression levels of β-catenin and TCF-4 as well as the TCF-4 promoter and TCF-mediated gene transcription activity were significantly higher than those in nontreated cells, but this did not happen in BE1 cells. We reasoned that this result had to do with differences in expression levels of endogenous Axin. The expression of endogenous Axin is relatively high in the A549 cell line, but relatively low in the BE1 cell line. Axin could provide a platform for GSK-3β facilitation of β-catenin degradation, thereby keeping β-catenin expression at a relatively low level. β-catenin expression was upregulated when LiCl blocked GSK-3β activity in A549 cells. Meanwhile, GSK-3β may not have been able to facilitate β-catenin degradation in BE1 cells because they lack the Axin platform. Likewise, LiCl treatment may also be unable to upregulate β-catenin expression further in BE1 cells by blocking GSK-3β activity. In addition, using GSK-3β siRNA could upregulate β-catenin and TCF-4 in A549 but not in BE1, which proved our hypothesis.

We previously reported that overexpression of Axin inhibits proliferative and invasive ability of lung cancer cells [18]. In this study, we found that transfected Axin and AxinΔp53 could inhibit the proliferative and invasive ability of BE1 and A549 cell lines significantly, but transfection of AxinΔβ-ca could only downregulate the proliferation and invasion of A549 cell significantly. Additionally, expression of AxinΔβ-ca also downregualtes proliferative and invasive ability significantly as powerful as AxinΔp53 in A549, which differ from their effects on TCF-4 transcription downregulation. This result indicated that Axin might inhibit proliferation and invasion via both β-catenin and wild type p53 pathway. This result indicated that Axin might inhibit proliferation and invasion via both β-catenin and wild type p53 pathway.

## Conclusions

In this study, we employed two lung cancer cell lines, BE1 (mutant p53) and A549 (wild-type p53) to delineate the mechanism by which Axin downregulates TCF-4 expression. We demonstrate that Axin downregulates TCF-4 transcription and TCF-mediated gene transcription in a β-catenin-dependent manner in lung cancer cells. We also show wild-type p53 induces of downregulation of TCF-4 transcription in lung cancer cells, and Axin coexpression does not lead to further inhibition. Axin downregulates TCF-4 transcription via the Axin-β-catenin-TCF-4 pathway. Axin might inhibit proliferation and invasion via both the β-catenin and p53 pathways. At the present time, there is much interest focused on the Wnt signaling pathway in cancer [29], yet research in this area has yielded little clinical impact [30]. Recently, we found that prognosis of patients with high expression of Axin was better than those with low expression, elevated Axin expression following X-ray exposure is a reliable indicator for determining the radiosensitivity of NSCLC[31]. Together, our study suggests that induction of Axin expression, in particular, has therapeutic potential for the treatment for lung cancer patients.

## Methods

### Plasmid construction

Mouse Axin cDNA (2.6 kb) was kindly provided by professor Perrella [32], and sequence verification was performed (TaKaRa Biotechnology Co., Japan). The Axin gene was inserted into the SacII and SmaI sites of the pEGFP-N1 vector (Clontech, Mountain View, CA), pEGFP-Axin, then used to construct Axin mutant vectors, pEGFP-AxinΔβ-ca (β-catenin binding site aa 433-501 mutation), pEGFP-AxinΔp53 (HIPK2 binding site aa 678-753 and p53 binding site aa 209-338 mutation), pEGFP-AxinΔβ&P (β-catenin, HIPK2 and p53 binding sites mutation). Deletion mutations were performed to construct these Axin mutant vectors (Figure 1A).

p53, β-catenin, GSK-3β siRNA sequences were purchased from Santa Cruz Biotechnology Inc. (Santa Cruz, CA). TCF-4 reporter gene construct, pGL-[1306]TCF4-Luc, were kindly provided by Dr. Kurt Engeland [33]. The TCF-mediated reporter gene construct, the wild type p53 plasmid, pGL3-OT (TopFlash) and pGL3-OF (FopFlash), were kindly provided by Dr. Bert Vogelstein [34].

### Cell Culture and Transfection

The BE1 cell line was a gift from Dr. Jie Zheng (College of Medicine, Beijing University, China). A549 cells were purchased from ATCC. Cells were cultured in RPMI 1640 medium (GIBCO Inc., Carlsbad, CA) and DMEM medium (GIBCO Inc.), respectively, with 10% fetal calf serum (GIBCO Inc.) at 37°C, 5% CO_2_.

BE1 and A549 cell were plated in 60 mm dishes. At approximately 90% confluency, cells were cultured for 3 h in medium without serum, and then transfected using Lipofectamine 2000 (Invitrogen, Carlsbad, CA) with empty vector or either mutant or wild type Axin according to the manufacturer's protocol. GSK-3β inhibitor, LiCl(20 mM) was added 24 h post-transfection [16].

### RT-PCR assay

Total RNA was extracted from samples using Trizol^® ^Reagent (Invitrogen, Carlsbad, CA). RT-PCR analysis was performed using primers specific for Axin, β-catenin, p53 and TCF-4 using a TaKaRa RNA PCR Kit (AMV) Ver. 3.0 (Takara, Dalian, China) according to the manufacturer's protocol. β-Actin served as an internal control. Primer sequences used are listed in Table 1. After resolving the products by agarose electrophoresis, the gel was stained with ethidium bromide and analyzed using a BioImaging system to visualize bands (UVP, Upland, CA). Relative band intensities were determined using NIH image software.

**Table 1:** Primer Sequences

name	primer sequence	product length	temperature	cycles
Axin*	Forward:5'-ACCGAAAGTACATTCTTGATAAC-3'	452	52	30
	Reverse:5'-TCCATACCTGAACTCTCTGC-3'			
Axin**	Forward:5'-TCCACCACCATGTTCACC-3'	243 bp	57.6°C	30
	Reverse:5'-CAGCATTGGCATTCTTCC-3'			
β-catenin	Forward:5'-GCCAAGTGGGTGGTATAGAG-3'	330 bp	49°C	30
	Reverse:5'-GCTGGGTATCCTGATGTGC-3'			
p53	Forward:5'-TCAGTCTACCTCCCGCCATA-3'	322 bp	58°C	30
	Reverse:5' TTACATCTCCCAAACATCCCT 3'			
TCF-4	Forward:5'-CGAGGGTGATGAGAACCTGC-3'	444 bp	52°C	30
	Reverse:5'-CCCATGTGATTCGATGCGT-3'			
β-actin	Forward:5'-AGAGCTACGAGCTGCCTGAC3'	308 bp	55°C	30
	Reverse:5'-AGTACTTGCGCTCAGGAGGA-3'			

* This primer specifically detects endogenous human Axin mRNA.

**This primer specifically detects exogenous mouse Axin mRNA, but not endogenous human Axin mRNA.

### Western blot assay

Cell pellets were collected and total protein was extracted using RIPA buffer + PMSF (Sigma-Aldrich, Inc., St. Louis, MO). Protein was quantified by staining with Coomassie Brilliant Blue G-250. Protein lysate was resolved by SDS-PAGE and subsequently transferred to PVDF membrane (Sigma-Aldrich, Inc.). Membrane was blocked in 3% fetal calf serum, followed by incubation with GFP antibody (1:200, Santa Cruz Biotechnology Inc, Santa Cruz, CA), Axin antibody (1:200, Santa Cruz Biotechnology Inc, Santa Cruz, CA), β-catenin antibody (1:800, BD Biosciences, San Jose, CA), mutant p53 antibody (DO-7, 1:200, Thermo Fisher Scientific Inc, Fremont, CA), wild type p53 antibody (Ab-5, 1:200, Calbiochem-Novabiochem Inc., La Jolla, CA), TCF-4 antibody (1:800, Millipore, USA), or β-actin antibody (1:200, Santa Cruz Biotechnology Inc.) overnight at 4°C. Membranes were washed and incubated with secondary antibody (Santa Cruz Biotechnology Inc.) at 37°C for 2 h. Membranes were washed and ECL reagent applied (Thermo Fisher Scientific Inc, Fremont, CA) and bands visualized by autoradiography (Kodak film KODAK, Japan). Protein bands were quantified by BioImaging Systems (UVP) as a ratio to β-actin expression in each sample.

### Dual-luciferase assay

Cells were co-transfected with TCF-4 promoter reporter gene plasmid, pGL-[1306]TCF4-Luc, or TCF-mediated transcription reporter gene plasmid, pGL3-OT and pGL3-OF, using Lipofectamine 2000 according to the manufacturer's instruction. Cells were plated in 24-well plates 24 h prior to transfection. After incubation for 48 h at 37°C, reporter gene expression was detected by the Dual-Luciferase Assay System (Promega, Madison, WI). TCF-mediated gene transcription activity was determined by the ratio of pGL3-OT to pGL3-OF luciferase activity, which was normalized to Renilla luciferase activity from the control plasmid, pRL-TK. TCF-4 promoter activity was determined by the value of pGL-[1306]TCF4-Luc luciferase activity, which was also normalized by Renilla luciferase activity of pRL-TK. The final relative luciferase activity was determined as the ratio of activity in cells transfected with Axin constructs to the EGFP transfected control cells.

### 3-(4,5-Dimethylthiazol-2-yl)-2,5-Diphenyltetrazolium Bromide (MTT) Assay

Cells were seeded in 96-well plates (1 × 10^4 ^cells/well) 12 h after transfection. Cells were counted each day for four days after transfection using the MTT method (MTT detection kit, Keygene, Nanjing, China) according to the manufacturer's instructions. The absorbance, which is directly proportional to the number of living cells in culture, was measured at 570 nm using a microplate reader (Model 550, Bio-Rad, Hercules, CA).

### Matrigel Invasive Assay

Cell invasive ability was examined using a 24-well transwell^® ^with 8 μm pore polycarbonate membrane inserts (Corning Inc., Lowell, MA) according to the manufacturer's protocol. Matrigel (100 μg/ml) was applied to the upper surface of the membranes. Cells were seeded on the upper chamber (5 × 10^4 ^cells/well) 24 h after transfection and incubated for 48 h. Cells that had invaded the surface of the membrane were fixed with methanol and stained with hematoxylin. Ten random high magnification microscope fields per filter were counted.

### Statistical Analysis

Statistics for the experimental data were performed by SPSS 13.0. Experiments were performed in triplicate, and the mean was calculated to be the final result. The Mann-Whitney U test was used to analyze the results of RT-PCR, Western blot, MTT, Matrigel invasive assay, TCF-4 promoter activity and TCF-mediated gene transcription activity. *P <*0.05 were considered statistically significant differences, *P <*0.01 were considered highly statistically significant differences.

## Competing interests

The authors declare that they have no competing interests.

## Authors' contributions

EH-W designed experiments and mentored the experimental process, LH-Y drafted the manuscript and YM, ZQ-Y performed the cell culture, transfection, RT-PCR and Western blot analyses, HT-X was responsible for Statistical Analysis, and plasmid, primer and experimental design. YH and QC-L participated in PCR analysis and data collection, and YL and YZ were responsible for the Dual-luciferase assay. QZ-D performed the MTT assay and Matrigel Invasive Assay, SD-D and CZ participated in the discussion, artwork and manuscript preparation. All authors have read and approved the final version of the manuscript.
